# Supplementary material for: An intronic transposon insertion associates with a trans-species color polymorphism in Midas cichlid fishes
Source: Nat Commun. 2022 Jan 13;13:296. doi: 10.1038/s41467-021-27685-8 (PMC8758764; doi:10.1038/s41467-021-27685-8)
Supplement: Supplementary file 1 — Supplementary Information [file 41467_2021_27685_MOESM1_ESM.pdf]

Supplementary materials

An intronic transposon insertion associates with a trans-species color polymorphism in Midas cichlid fishes

Claudius F. Kratochwil[1,2], Andreas F. Kautt[1,3], Alexander Nater[1], Andreas Härer[1,4], Yipeng Liang[1,5], Frederico Henning[6] and Axel Meyer[1]

[1] Zoology and Evolutionary Biology, Department of Biology, University of Konstanz, Universitätsstrasse 10, 78457 Konstanz, Germany

[2] Present address: Institute of Biotechnology, HiLIFE, University of Helsinki, Helsinki, Finland

[3] Present address: Department of Organismic and Evolutionary Biology, Harvard University, Cambridge MA 02138, U.S.A.

[4] Present address: Division of Biological Sciences, Section of Ecology, Behavior & Evolution, University of California San Diego, La Jolla CA, U.S.A.

[5] Present address: Department of Biology, University of Virginia, Charlottesville VA 22903, U.S.A.

[6] Department of Genetics, Institute of Biology, Federal University of Rio de Janeiro (UFRJ), Rio de Janeiro, Brazil.

Corresponding authors:

Claudius F. Kratochwil, Institute of Biotechnology, HiLIFE, University of Helsinki, Helsinki, Finland. E-mail: Claudius.Kratochwil@helsinki.fi

Axel Meyer, Department of Biology, University of Konstanz, Konstanz 78457, Germany. E-mail: Axel.Meyer@uni-konstanz.de

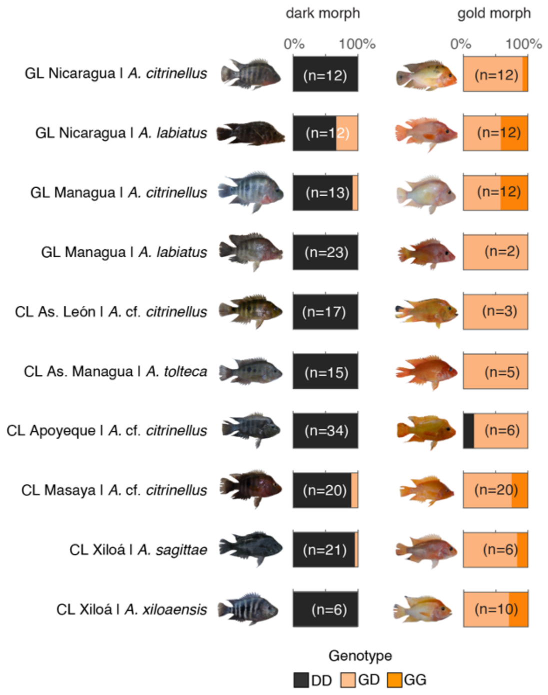

Supplementary Figure 1 | Genotype distributions across polymorphic lakes and species. Summary of all species and populations with their respective phenotypes and genotypes at the transposon. Please note that for CL Xiloá twelve (dark) hybrids between *A. sagittae* and *A. xiloaensis* are not shown (all have the *dd* genotype).

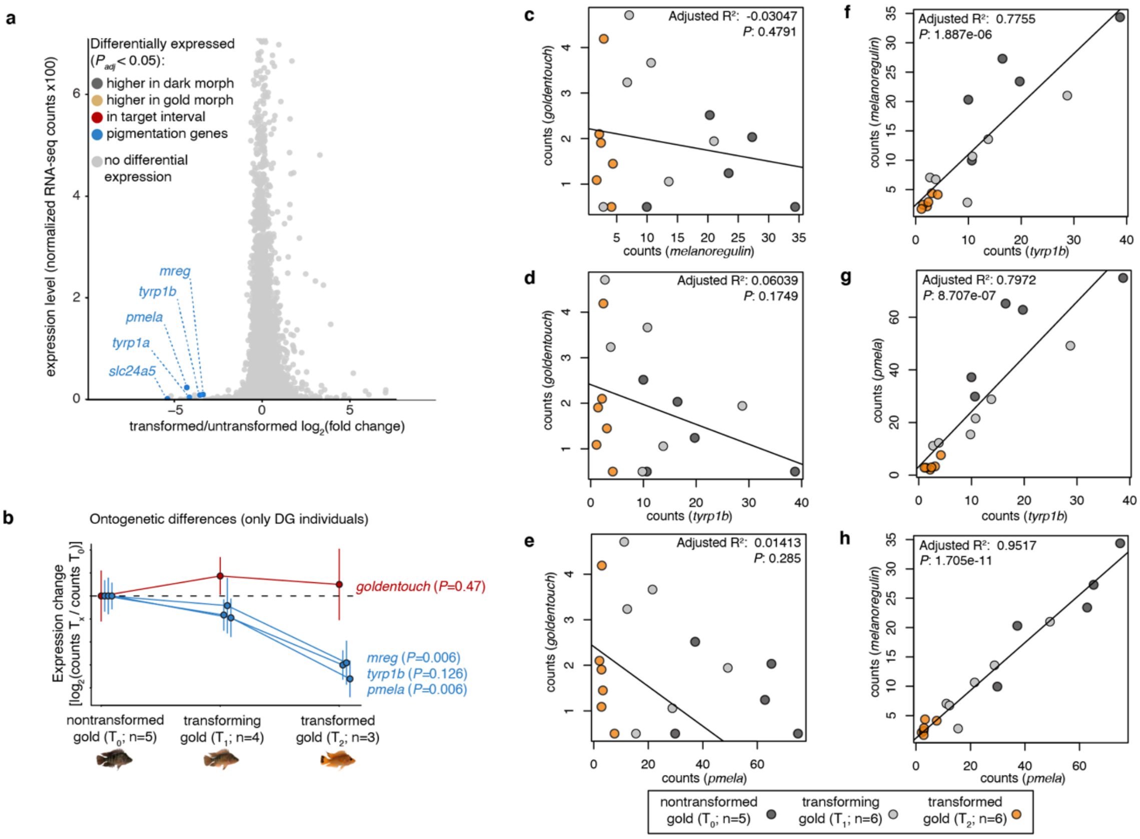

Supplementary Figure 2 | Additional RNA-seq analysis. a, Reanalysis of a previous RNA-seq study on scales of gold fish at different ontogenetic stages (here comparison between gene expression of genotypically golden individuals before and after transition) confirm five differentially expressed genes between transformed and untransformed genetically gold individuals (P_{adj}<0.05; Benjamini-Hochberg adjusted Wald test P-value). Interestingly and in contrast to the comparison beween genetically dark and gold individuals these genes only include pigmentation genes (*slc24a5*, P_{adj} = 0.0019; *tyrp1a*, P_{adj} = 0.0056; *mreg*, P_{adj} = 4.26E-06; *pmela*, P_{adj} = 1.36E-14; *tyrp1b*, P_{adj} = 0.0008). **b,** Same analysis as Fig. 2f, but only using individuals with the genotype Gd (data are presented as mean values +/- SD; statistical test: one-way ANOVA). **c-h,** Correlation between normalized RNA-seq counts of goldentouch and pigmentation genes (c-e; no correlation) and between pigmentation genes (f-h, strong correlation), suggesting that goldentouch expression is not in melanophores itself and varies between genetically gold and dark individuals but not between stages (before and after transition) of genetically gold individuals (Adjusted R-squared and P-value of linear regression).

Supplementary Table 1 | Enriched gene ontology (GO) terms. List of enriched gene ontology terms based.

Enrichment FDR	Genes in list	Total genes	Functional Category
2.82E-13	21	302	Extracellular space
4.50E-12	36	1266	Extracellular region
8.46E-07	7	43	Purine nucleoside diphosphate metabolic process
8.46E-07	7	43	Purine ribonucleoside diphosphate metabolic process
8.46E-07	7	43	Ribonucleoside diphosphate metabolic process
8.46E-07	4	5	Melanin biosynthetic process
8.46E-07	4	5	Secondary metabolite biosynthetic process
8.46E-07	7	41	ADP metabolic process
8.46E-07	7	43	Pyruvate metabolic process
8.46E-07	7	38	Glycolytic process
8.46E-07	7	38	ATP generation from ADP
9.15E-07	8	68	Negative regulation of endopeptidase activity
1.37E-06	7	47	Carbohydrate catabolic process
1.50E-06	23	945	Proteolysis
1.57E-06	4	6	Secondary metabolic process
1.57E-06	4	6	Melanin metabolic process
1.73E-06	11	190	Purine ribonucleotide metabolic process
1.73E-06	8	77	Regulation of endopeptidase activity
1.73E-06	50	3704	Organonitrogen compound metabolic process
1.74E-06	8	79	Negative regulation of peptidase activity
1.74E-06	8	79	Negative regulation of proteolysis
2.02E-06	7	54	Nucleotide phosphorylation
2.02E-06	11	196	Purine nucleotide metabolic process
2.02E-06	7	54	Nucleoside diphosphate phosphorylation
2.87E-06	11	205	Ribonucleotide metabolic process
2.91E-06	11	206	Ribose phosphate metabolic process
2.98E-06	7	58	Nucleoside diphosphate metabolic process
3.13E-06	11	209	Purine-containing compound metabolic process
4.59E-06	8	93	Regulation of peptidase activity
6.46E-06	8	98	Endopeptidase inhibitor activity
