# Supplementary material for: An intronic transposon insertion associates with a trans-species color polymorphism in Midas cichlid fishes
Source: Nat Commun. 2022 Jan 13;13:296. doi: 10.1038/s41467-021-27685-8 (PMC8758764; doi:10.1038/s41467-021-27685-8)
Supplement: Supplementary file 3 — Description of Additional Supplementary Files [file 41467_2021_27685_MOESM3_ESM.pdf]

Description of Additional Supplementary Files

File Name: Supplementary Data 1
Description: List of differentially expressed genes.
