# Supplementary material for: An intronic transposon insertion associates with a trans-species color polymorphism in Midas cichlid fishes
Source: Nat Commun. 2022 Jan 13;13:296. doi: 10.1038/s41467-021-27685-8 (PMC8758764; doi:10.1038/s41467-021-27685-8)
Supplement: Supplementary file 5 — Reporting Summary [file 41467_2021_27685_MOESM5_ESM.pdf]

nature research

Corresponding author(s): Claudius Kratochwil, Axel Meyer

Last updated by author(s): Nov, 11 2021

Reporting Summary

Nature Research wishes to improve the reproducibility of the work that we publish. This form provides structure for consistency and transparency in reporting. For further information on Nature Research policies, see our Editorial Policies and the Editorial Policy Checklist.

Statistics

For all statistical analyses, confirm that the following items are present in the figure legend, table legend, main text, or Methods section.

n/a	Confirmed	
[ ]	[x]	The exact sample size (*n*) for each experimental group/condition, given as a discrete number and unit of measurement
[x]	[ ]	A statement on whether measurements were taken from distinct samples or whether the same sample was measured repeatedly
[ ]	[x]	The statistical test(s) used AND whether they are one- or two-sided<br>*Only common tests should be described solely by name; describe more complex techniques in the Methods section.*
[x]	[ ]	A description of all covariates tested
[x]	[ ]	A description of any assumptions or corrections, such as tests of normality and adjustment for multiple comparisons
[ ]	[x]	A full description of the statistical parameters including central tendency (e.g. means) or other basic estimates (e.g. regression coefficient) AND variation (e.g. standard deviation) or associated estimates of uncertainty (e.g. confidence intervals)
[ ]	[x]	For null hypothesis testing, the test statistic (e.g. *F*, *t*, *r*) with confidence intervals, effect sizes, degrees of freedom and *P* value noted<br>*Give P values as exact values whenever suitable.*
[x]	[ ]	For Bayesian analysis, information on the choice of priors and Markov chain Monte Carlo settings
[x]	[ ]	For hierarchical and complex designs, identification of the appropriate level for tests and full reporting of outcomes
[x]	[ ]	Estimates of effect sizes (e.g. Cohen's *d*, Pearson's *r*), indicating how they were calculated

Our web collection on statistics for biologists contains articles on many of the points above.

Software and code

Policy information about availability of computer code

Data collection	no software was used for data collection
Data analysis	Improved Phased Assembler (IPA) v.1.3.0, GEMMA v0.98.1, trimmomatic v0.38, STAR RNA-seq aligner v2.6.1d, RSEM v.1.3.3, DESEQ2 v1.22.1, MAFFT v7.407, BMGE v1.12. , Mr. Bayes v.3.2.6, ShinyGO v.0.66, Protein Homology/analogY Recognition Engine V 2.0 (Phyre2), trRosetta (web version July 2021)

For manuscripts utilizing custom algorithms or software that are central to the research but not yet described in published literature, software must be made available to editors and reviewers. We strongly encourage code deposition in a community repository (e.g. GitHub). See the Nature Research guidelines for submitting code & software for further information.

Data

Policy information about availability of data

All manuscripts must include a data availability statement. This statement should provide the following information, where applicable:

- Accession codes, unique identifiers, or web links for publicly available datasets
- A list of figures that have associated raw data
- A description of any restrictions on data availability

RNA-seq raw reads (PRJNA635556, PRJNA766232), Pac-Bio raw reads (PRJNA694028) are available at the Sequence Read Archive. PacBio assembly, sample lists and intermediate data are available at Dryad (https://doi.org/10.5061/dryad.zs7h44j9p).

Field-specific reporting

Please select the one below that is the best fit for your research. If you are not sure, read the appropriate sections before making your selection.

- [x] Life sciences
- [ ] Behavioural & social sciences
- [ ] Ecological, evolutionary & environmental sciences

For a reference copy of the document with all sections, see nature.com/documents/nr-reporting-summary-flat.pdf

Life sciences study design

All studies must disclose on these points even when the disclosure is negative.

Sample size	Sample sizes for the genomic part are n=273 (88 gold, 185 dark). For the RNA-seq experiment n=18 (9 per morph), for the qPCR n=12 (n=6 per morph). For the genomic part we used all available genomic information. For the RNA-seq experiment we used a relatively high sample size (normally we use 6) to obtain robust data. Also, we wanted to have at least 3 samples per morph from three different populations. N=6 is the standard sample size we are using for qPCR based on previous studies.
Data exclusions	No data was excluded
Replication	For qPCR we used technical (three and two technical replicates for target genes and housekeeping genes, respectively — we use mean threshold cycle (Ct) values of those) and biological replicates. Sample sizes for the other experiments are stated above. Genomic and RNA-seq experiments have been only performed once.
Randomization	Within groups (i.e., morphs) individuals included in this study were sampled randomly. Groups were defined based on morph. All subsequent analysis were performed an all samples, hence no randomization was necessary.
Blinding	All phenotypic assignment were repeated by three different observers, without any deviation in phenotype assessment.

Reporting for specific materials, systems and methods

We require information from authors about some types of materials, experimental systems and methods used in many studies. Here, indicate whether each material, system or method listed is relevant to your study. If you are not sure if a list item applies to your research, read the appropriate section before selecting a response.

Materials & experimental systems

n/a	Involved in the study	
x		Antibodies
x		Eukaryotic cell lines
x		Palaeontology and archaeology
	x	Animals and other organisms
x		Human research participants
x		Clinical data
x		Dual use research of concern

Methods

n/a	Involved in the study	
x		ChIP-seq
x		Flow cytometry
x		MRI-based neuroimaging

Animals and other organisms

Policy information about studies involving animals; ARRIVE guidelines recommended for reporting animal research

Laboratory animals	Laboratory animals were bred and raised in the animal research facility of the University of Konstanz. All animals were kept at a constant 12:12 light-dark cycle and at a constant temperature of 28 degrees celsius. At no time were fish held on any kind of caloric restrictions.
Wild animals	This study did not involve experimentation on wild animals.
Field-collected samples	No new field-collected samples were part of this study. The analysis of field-collected samples was based on previously published data (Kautt et al. Nature 2020; PRJEB38173).
Ethics oversight	Euthanization of animals and animal husbandry were approved by the German authorities (permit numbers T-16/13, Regierungspräsidium Freiburg, Abteilung 3, Referat 35, Veterinärwesen & Lebensmittelüberwachung, Germany).

Note that full information on the approval of the study protocol must also be provided in the manuscript.
